# Can the Use of Telehealth Guidance Services Reduce Depressive Symptoms Among Family Caregivers of Older Adults with Cognitive Impairment? A Moderated-Mediation Model

**DOI:** 10.3390/healthcare14091234

**Published:** 2026-05-03

**Authors:** Li Li, Hao Zhou, Xiaorong Gao, Keke Chen, Qiaoqiao Wang

**Affiliations:** 1 School of Medicine, Hangzhou City University, Hangzhou 310015, China; lili@hzcu.edu.cn (L.L.);; 2 Hangzhou Binjiang District Center for Disease Control and Prevention, Hangzhou 310051, China

**Keywords:** telehealth guidance services, caregivers, depressive symptoms, caregiving competence, resilience

## Abstract

**Background:** Family caregivers of older adults with cognitive impairment commonly encounter heavy care burdens and elevated mental health risks, particularly depressive symptoms. This study aimed to explore the association between telehealth guidance service use and depressive symptoms among family caregivers of older adults with cognitive impairment, and to further examine the mediating role of caregiving competence and the moderating role of psychological resilience. **Methods:** A cross-sectional survey of 491 family caregivers of older adults with cognitive impairment was conducted from August to October 2023. Descriptive statistics, correlation analysis, linear regression analysis, and moderated-mediating-effect analysis were employed. **Results:** Among the participants, only 17.31% reported using telehealth guidance services. Mean scores for caregiving competence, psychological resilience, and depressive symptoms were 3.04 ± 0.48, 27.11 ± 7.54, and 9.69 ± 1.46, respectively. Telehealth service use was positively associated with caregiving competence, and both telehealth service use and caregiving competence were negatively associated with depressive symptoms. The interaction between psychological resilience and caregiving competence was also significantly negatively associated with depressive symptoms (*p* < 0.01). **Conclusions:** Telehealth guidance service use is directly and indirectly negatively associated with depressive symptoms via caregiving competence. Psychological resilience moderates the relationship between caregiving competence and depressive symptoms. These findings contribute to a better understanding of factors linked to mental health among family caregivers of older adults with cognitive impairment.

## 1. Introduction

As the global population ages, the prevalence of cognitive impairment is expected to rise, with advanced age being a major risk factor. China, which has the world’s largest number of individuals with cognitive impairment, accounts for approximately 25% of cases worldwide [1]. Cognitive impairment, a complex, progressive, and largely irreversible neurodegenerative disorder, features persistent declines in cognitive, emotional, and physical functional abilities [2]. These declines lead these older adults to be dependent on others and in need of sustained care. Rooted in traditional Confucian values of filial piety and hindered by scarce formal care resources, roughly 90% of Chinese older adults with cognitive impairment receive home-based care from family members [3]. These family caregivers play an essential yet highly demanding role. In addition to providing extensive support for activities of daily living, they must also manage the behavioral and psychological symptoms of cognitive impairment [4,5]. The heavy burden of long-term care work leads many family caregivers to experience fatigue, negative psychological reactions, depression, and other issues.

Existing research has demonstrated that the prevalence of depressive symptoms among dementia caregivers is significantly higher than that among general older adult caregivers [6]. Thus, developing scientific and effective interventions in this vulnerable population carries substantial theoretical and practical significance.

Depression among family caregivers of individuals with cognitive impairment is influenced by multiple factors, including the demographic and clinical factors of the care recipient, caregiving competence and tasks, caregivers’ personality traits, and social support [7]. These factors interact with each other, among which family caregiving competence is a relatively core factor. However, numerous studies have shown that family caregivers generally face the dilemma of lacking related knowledge, essential caregiving skills, stress management abilities, communication skills, and social support resources [8,9]. Drawing on Empowerment Theory, first proposed by Solomon in the 1970s in social psychology [10], multicomponent strategies that integrate educational, physical, psychological and emotional support may be associated with addressing the evolving needs of family caregivers. Such supports correlate with better self-efficacy, favorable mental well-being, and lower emotional distress.

Although numerous traditional interventions, including printed educational guides, peer support groups, and in-person workshops, have been widely implemented and shown to yield certain beneficial effects, such approaches are often limited in population coverage and operational flexibility [11].

In this context, digital and intelligent technologies have become promising means to empower family caregivers and improve their caregiving competence. Through remote health guidance, online diagnosis and treatment, and virtual reality-enabled targeted rehabilitation training, these technologies may be directly positively associated with health status among individuals with cognitive impairment. Meanwhile, intelligent monitoring tools, including wearable devices and smart dynamic health monitoring platforms, enable timely risk detection and early intervention, thereby alleviating the daily care burden of family caregivers and relieving the excessive physical and mental strain associated with long-term caregiving [12,13,14]. Additionally, digital mobile health applications and platforms can offer family caregivers tailored services such as remote training and online consultation, allowing them to systematically acquire professional care knowledge, master core caregiving skills, and strengthen their caregiving capacity [15,16].

Previous studies have shown that digital and intelligent technology empowerment is positively associated with caregiving competence. Other research has also demonstrated that caregiving competence is associated with caregivers’ subjective burden, as well as lower levels of anxiety and depression. However, the relationship between intelligent technology use, caregiving competence, and depressive symptoms remains underexplored. Accordingly, the following hypotheses are proposed in this study: Hypothesis 1: The use of a telehealth guidance service is positively associated with caregiving competence. Hypothesis 2: The use of a telehealth guidance service is negatively associated with depressive symptoms. Hypothesis 3: Caregiving competence mediates the negative association between the use of a telehealth guidance service and depressive symptoms.

Notably, existing research has demonstrated that caregivers with similar levels of caregiving competence may still present significant disparities in mental health outcomes, implying the potential influence of unmeasured moderating variables. Currently, the Chinese government has paid increasing attention to family caregivers and implemented multiple measures to improve their caregiving abilities. However, available social resources remain limited, so targeted interventions tailored to individual characteristics are essential to enhance caregiving capacity and relieve poor mental conditions. In this context, identifying valid moderating factors is of great practical significance. As a critical internal psychological resource that facilitates adaptation to adversity, psychological resilience is widely recognized as an important protective factor that buffers stress and moderates emotional responses. Empirical studies have revealed that among caregivers of stroke, epilepsy, and dementia patients, psychological resilience is significantly and positively correlated with quality of life and negatively correlated with depressive symptoms [17]. Wang reported that psychological resilience is negatively associated with depressive symptoms and may also serve as a significant moderator in the relationship between adverse events and depressive symptoms [18]. Therefore, Hypothesis 4 is proposed: Psychological resilience plays a moderating role between caregiving competence and depressive symptoms.

## 2. Materials and Methods

### 2.1. Sample

A cross-sectional survey was conducted in Hangzhou, Zhejiang Province, China, from August to October 2023. A multistage stratified sampling method was adopted to select 30 communities across 4 districts. With the assistance of staff from the sampled subdistricts, questionnaire surveys were administered to families with members aged 60 years and older. The questionnaires were completed independently by the elderly individuals (≥60 years old) and their main family caregivers.

Family caregivers were required to satisfy all of the following inclusion criteria: (1) being aged 18 years or older and possessing full civil capacity; (2) being a member of the care recipient’s family and assuming primary responsibility for their daily living care; (3) demonstrating mental clarity and the ability to accurately understand and report basic family conditions and current care status. Each questionnaire was accompanied by a detailed information sheet that described the study aims, research procedures, ethical considerations, and specific guidance for completion. A total of 1265 valid questionnaires were collected from family caregivers.

Since this study focused primarily on the family care context of older adults with cognitive impairment, only families with care recipients meeting any one of the following criteria were included in the data analysis: (1) the care recipient had been formally diagnosed with cognitive impairment by a hospital of any level or identified as having cognitive impairment through screening at a primary medical institution; (2) the care recipient was unable to complete the questionnaire independently during the survey due to severe cognitive impairment symptoms; (3) the care recipient scored less than 26 on the Montreal Cognitive Assessment (MoCA), which is the internationally recognized cutoff for cognitive impairment screening. According to the above criteria, a final total of 491 valid participants were included in the statistical analysis.

### 2.2. Measures

#### 2.2.1. Family Caregivers’ Caregiving Competence

The family caregivers’ caregiving competence was assessed using a self-developed scale. Twenty-three items related to caregiving competence were developed through intensive qualitative interviews with policymakers, professional caregivers, family caregivers for individuals with cognitive impairment, and experts in the field and an initial pilot study [19,20]. Respondents were required to assess their perception of caregiving competence on each item based on a 5-point Likert scale (1 = very weak; 2 = slightly weak; 3 = somewhat average; 4 = strong; and 5 = extremely strong). A subsequent factor analysis was conducted using principal component analysis with varimax rotation. In the initial stage, we examined the factor loadings of all items. Three incentive items were removed because they had factor loadings of less than 0.4, or they showed nearly equal loadings on two different sub-scales. After the removal of these items, a second factor analysis was performed. As a result, four distinct and interpretable sub-scales comprising 20 items were successfully identified. Based on the conceptual meanings of the items and the version of the Family Caregiving Competence Scale for Elderly Patients revised by Jin et al. and the Caregiving Competence Scale for caregivers of individuals with cognitive impairment developed by Xie et al., these four sub-scales of family caregiving competence were named caregiving cognitive ability, daily living and nursing skills, family cohesion, and resource integration ability, and individually, they accounted for 34.62%, 16.99%, 9.09%, and 5.05% of the overall variance, respectively. The mean caregiving competence score was calculated as the total score of the 20 items divided by 20. The Cronbach’s α coefficient for the scale was 0.90, demonstrating good internal consistency and reliability.

#### 2.2.2. Psychological Resilience

Psychological resilience was assessed using the 10-item Chinese version of the Connor–Davidson Resilience Scale (CD-RISC-10). The original 25-item scale was developed by Connor and Davidson, encompassing five dimensions. Based on this version, Campbell-Sills et al. later developed and validated a 10-item short form [21], which was subsequently translated and validated in Chinese by Zhang et al. [22]. The Chinese version comprises two dimensions: strength and tenacity. The scale uses a 5-point Likert format, with responses ranging from 0 (never) to 4 (almost always). Total scores were calculated as the sum of all items, ranging from 0 to 40, where higher scores indicate greater psychological resilience. The Cronbach’s α coefficient for the scale was 0.87. For the moderating effect analysis in this study, the total score was recoded into a dichotomous variable using a cutoff score of 20: participants with a total score ≤ 20 were classified into the low-resilience group (coded 1), while those with a score > 20 were assigned to the high-resilience group (coded 2).

#### 2.2.3. Use of Telehealth Guidance Services

For the purpose of this study, the use of telehealth guidance services is defined as access to and use of cognitive impairment-specific professional supportive interventions delivered through digital and remote communication modalities by family caregivers of older adults with cognitive impairment. Based on a systematic literature review and expert consultation, this study assessed the use of four main types of telehealth guidance: telemedicine support (remote medical consultation and professional health guidance for care recipients), specialized care training (remote skill training for daily care or behavior management of cognitive-impaired older adults), one-on-one professional consultation (targeted problem-solving guidance for caregiving dilemmas and psychological support), and remote care monitoring assistance (basic health status monitoring and early warning support for care recipients) [23,24,25]. The respondents were asked to report whether they had used any of these targeted professional telehealth guidance services via digital or remote communication tools at any point within the past 6 months. The variable was coded as a binary indicator, where respondents who selected “Yes” to any one or more of the four telehealth service items were coded as 1, and those who selected “No” across all four items were coded as 0.

#### 2.2.4. Depressive Symptoms

In this study, depressive symptoms among family caregivers of older adults with cognitive impairment were assessed using the short-form version of the Center for Epidemiological Studies Depression Scale (CES-D). This scale asks respondents to rate the frequency of symptoms over the past week. It consists of 8 items assessing negative emotions, with each item scored from 0 to 3 according to how often the symptom occurred. Two items measuring positive emotions were reverse-coded. Total scores ranged from 0 to 30, with higher scores indicating more severe depressive symptoms [26]. The Cronbach’s α coefficient for this scale in the present study was 0.92.

#### 2.2.5. Cognitive Status of Care Recipients

The cognitive function of care recipients was assessed using the Montreal Cognitive Assessment (MoCA). The scale covers eight domains: visuospatial/executive function, naming, memory (not scored), attention, language, abstraction, delayed recall, and orientation. The total score ranges from 0 to 30, with higher scores indicating better cognitive function. A score of ≥26 was classified as normal cognition, 18–25 as mild cognitive impairment (MCI), 10–17 as moderate cognitive impairment, and <10 as severe cognitive impairment. For participants with ≤12 years of education (high school level or below), 1 point was added to the raw score (not exceeding 30) to adjust for educational bias.

### 2.3. Statistical Analysis

All data in this study were analyzed using SPSS software (Version 25.0). Descriptive statistics were first employed to characterize the sociodemographic characteristics, caregiving status, family caregiving competence, and use of telehealth guidance services among family caregivers. Second, independent-samples *t*-tests and one-way ANOVA were performed to examine group differences in depressive symptoms. Variables showing statistically significant differences were then included as control variables in the subsequent moderated-mediation model. Spearman’s rank correlation analysis was used to explore the correlations between telehealth guidance service use, caregiving competence, psychological resilience and depressive symptoms. Hayes’ PROCESS Model 4 was then employed to examine the mediating effect of caregiving competence, in which family caregiving competence acted as a mediator. Subsequently, PROCESS Model 14 was applied to test the moderated-mediation model, in which family caregiving competence acted as a mediator and psychological resilience served as a moderator in the relationship between telehealth guidance service use and depressive symptoms. A bootstrap resampling method with 5000 iterations was adopted to verify the significance of the mediating and moderating effects; statistical significance was determined if the 95% confidence interval (CI) of the effect size did not include zero.

## 3. Results

### 3.1. Fundamental Characteristics of Respondents

The sociodemographic and caregiving characteristics of the study sample are presented in Table 1. Most caregivers were female (61.30%), aged 45–59 years (58.04%), with junior college or above education (35.44%). Among caregivers, 83.51% reported a daily caregiving duration of ≤8 h, and 55.39% provided care 1–2 days per week. Regarding cognitive impairment in care recipients, 64.97% had mild impairment, 29.53% moderate, and 5.50% severe.

Group comparisons revealed significant differences in depressive symptoms by age, monthly income, daily care hours, weekly care days, and recipient cognitive severity (*p* < 0.01). No significant differences were found by gender or education (*p* > 0.05).

### 3.2. Descriptive Statistics and Correlations Between Variables

Descriptive statistics and bivariate correlations between the main variables are presented in Table 2. Among 491 family caregivers of older adults with cognitive impairment, 85 (17.31%) reported using telehealth guidance services. The mean score was 3.04 ± 0.48 for caregiving competence, 27.11 ± 7.54 for psychological resilience, and 9.69 ± 1.46 for depressive symptoms.

Bivariate correlation analyses showed that telehealth guidance service use was positively associated with caregiving competence (r = 0.218, *p* < 0.01) and psychological resilience (r = 0.154, *p* < 0.01), and negatively associated with depressive symptoms (r = −0.337, *p* < 0.01). Caregiving competence was negatively associated with depressive symptoms (r = −0.474, *p* < 0.01), and psychological resilience was also negatively associated with depressive symptoms (r = −0.317, *p* < 0.01). All correlation results were consistent with the hypothesized directions and supported the conduct of subsequent mediation analysis.

### 3.3. Mediating Effect of Caregiving Competence in the Relationship Between Telehealth Guidance Service Use and Depressive Symptoms

A mediation model was conducted using Model 4 in the SPSS PROCESS macro, with telehealth guidance service use as the independent variable (X), caregiving competence as the mediator (M), and depressive symptoms as the dependent variable (Y). Age, monthly family income, daily caregiving duration, weekly caregiving days, and care recipient cognitive impairment severity were included as control variables. Bootstrap resampling (5000 samples) was applied to obtain 95% bias-corrected confidence intervals.

As presented in Table 3, telehealth guidance service use was negatively associated with depressive symptoms (β = −0.641, *t* = −4.669, *p* < 0.01) and positively associated with caregiving competence (β = 0.389, *t* = 3.113, *p* < 0.01). Further, in the hierarchical model adjusting for caregiving competence, telehealth guidance service use remained negatively associated with depressive symptoms (β = −0.474, t = −3.709, *p* < 0.01), and caregiving competence was negatively associated with depressive symptoms (β = −0.430, t = −9.341, *p* < 0.01).

As shown in Table 4, the total association between telehealth guidance service use and depressive symptoms was −0.641 (SE = 0.137, 95% CI: −0.911, −0.371). The direct association was −0.474 (SE = 0.128, 95% CI: −0.725, −0.223), accounting for 73.95% of the total association. The indirect association via caregiving competence was −0.167 (SE = 0.055, 95% CI: −0.283, −0.064), accounting for 26.05% of the total association.

Because the 95% confidence interval for the indirect association did not include zero, caregiving competence exhibited a significant partial mediating role in the relationship between telehealth guidance service use and depressive symptoms.

### 3.4. Moderated-Mediation Model with Psychological Resilience as a Moderator

Based on the significant mediating effect identified above, a moderated-mediation model (Model 14, SPSS PROCESS macro) was examined to test whether psychological resilience moderated the second stage of the mediating pathway (i.e., the association between caregiving competence and depressive symptoms). Control variables were identical to those in the mediation analysis.

As shown in Table 5, in Regression 1 (outcome: caregiving competence), telehealth guidance service use was positively associated with caregiving competence (β = 0.389, SE = 0.125, t = 3.113, *p* < 0.01). In Regression 2 (outcome: depressive symptoms), caregiving competence (β = −0.366, SE = 0.051, t = −7.228, *p* < 0.01) and psychological resilience (β = −1.193, SE = 0.375, t = −3.180, *p* < 0.01) were negatively associated with depressive symptoms. The interaction term between caregiving competence and psychological resilience was significantly negatively associated with depressive symptoms (β = −0.230, SE = 0.106, t = −2.172, *p* < 0.05), while telehealth guidance service use remained significantly negatively associated with depressive symptoms (β = −0.417, SE = 0.127, t = −3.292, *p* < 0.01). These results confirmed that psychological resilience significantly moderated the second stage of the mediating pathway.

To further clarify the nature of this moderation, simple slope analysis and conditional indirect effect tests were conducted. Low and high psychological resilience were defined as scores below and above the mean value, respectively, consistent with the operationalization described in the Section 2.

**Figure 1:**
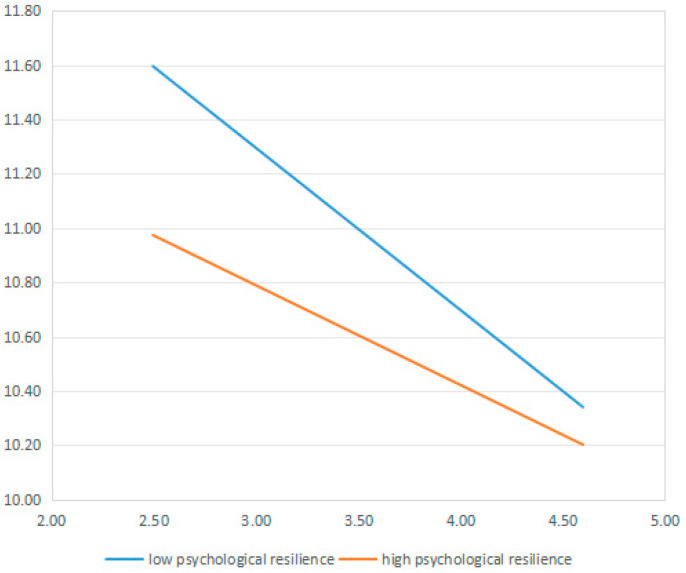
Moderating effect of psychological resilience on the association between caregiving competence and depressive symptoms.

As shown in Table 6, caregiving competence was negatively associated with depressive symptoms at both levels of psychological resilience. However, the strength of this negative association differed significantly by resilience level. Specifically, the negative association was stronger among caregivers with low psychological resilience (β = −0.233, 95% CI: −0.406, −0.087) than among those with high psychological resilience (β = −0.143, 95% CI: −0.245, −0.053). As visually presented in Figure 1, the slope depicting the relationship between caregiving competence and depressive symptoms was notably steeper for caregivers with low psychological resilience, whereas the slope was relatively milder for those with high psychological resilience. This further supports that the negative association between caregiving competence and depressive symptoms was stronger at lower levels of psychological resilience.

## 4. Discussion

### 4.1. Overall Characteristics of Telehealth Guidance Service Use, Caregiving Competence, Psychological Resilience, and Depressive Symptoms

Our findings revealed that the utilization rate of telehealth guidance service among family caregivers of older adults with cognitive impairment was relatively low (17.31%). This observation aligns with previous studies, which similarly report limited adoption of telehealth interventions among this vulnerable population [27,28]. Several factors may be associated with this low uptake. First, the digital divide acts as a significant obstacle. Many family caregivers in this group are elderly and often possess limited digital literacy. They may struggle with basic digital operations, and this lack of digital skills may directly impact their engagement with telehealth platforms [29,30]. Second, many telehealth guidance services are not tailored to the unique needs of this caregiving population. The absence of scenario-based adaptation and the presence of fragmented service delivery result in a notable mismatch between provided contents and caregivers’ actual demands [31,32]. Third, growing risks and ethical concerns regarding the privacy protection and data security of personal health and medical information further hinder long-term acceptance and sustained use of digital health tools [32].

This study also assessed depressive symptom levels and the associated factors among these family caregivers. Specifically, higher depressive symptom levels were observed among caregivers who were older, had lower family incomes, spent more hours on daily care, provided care more frequently each week, or cared for individuals with more severe cognitive impairment. These findings are consistent with previous research [33,34]. Notably, no significant gender difference in depressive symptoms was found in this study, which differs from many prior studies reporting higher depressive symptoms among female caregivers [35]. The inconsistency may be related to variations in sample characteristics, cultural backgrounds, and measurement tools, and thus warrants further investigation.

In addition, participants in this study demonstrated moderate levels of psychological resilience (M = 27.11, SD = 7.54), which is consistent with evidence from international studies of dementia caregivers [36,37]. With respect to caregiving competence, the mean score was 3.04 ± 0.48, indicating an overall moderate level. This result is consistent with the existing literature reporting suboptimal caregiving competence among family caregivers of persons living with cognitive impairment [9].

### 4.2. Relationships Between Telehealth Guidance Service Use, Caregiving Competence, and Depressive Symptoms

The present findings indicate that telehealth guidance service use was directly negatively associated with caregivers’ depressive symptoms, and also indirectly associated with lower depressive symptoms through caregiving competence, supporting the mediating role of caregiving competence. This pattern is consistent with the existing literature. For instance, Au et al. reported that a telephone-based psychoeducation and behavioral activation intervention was negatively associated with depressive symptoms among family caregivers of individuals with Alzheimer’s dementia [38]. In a systematic review and meta-analysis, Scerbe A noted that digital education interventions were related to lower depressive symptoms in caregivers, both directly and indirectly through improved caregiving skills [39].

A range of telehealth guidance strategies is available, including skills training and coaching, professional consultation, decision support, and remote monitoring. As highlighted by Lerman et al., remote training via video conferencing provides a feasible approach for implementing behavioral interventions, enabling real-time feedback and care skill modeling from professional practitioners [40]. Similarly, González et al. observed that training programs were positively associated with practical care management skills, while tailored consultation was positively linked to performance in complex care-related decision-making [41].

### 4.3. Moderating Role of Psychological Resilience in the Relationship Between Caregiving Competence and Depressive Symptoms

The present findings demonstrated that psychological resilience was negatively associated with depressive symptoms, which aligns with most previous studies [42]. Meanwhile, the interaction term between psychological resilience and caregiving competence was significantly negatively associated with depressive symptoms, supporting the moderating role of psychological resilience. These results are in line with Wang et al., who also identified negative associations between psychological resilience and depressive symptoms, as well as a significant moderating effect among older adults who suffered the falls [18,43].

Simple slope analyses and conditional indirect effect tests further supported the presence of significant moderation. The negative association between caregiving competence and depressive symptoms was significantly stronger among caregivers with lower psychological resilience than among those with higher levels. These findings indicate that low psychological resilience may amplify the inverse association between caregiving competence and depressive symptoms.

This moderating pattern can be interpreted from the perspective of resource substitution theory. Individuals with higher psychological resilience tend to show greater adaptability in stressful caregiving situations, and their lower depressive symptoms are often accompanied by stronger cognitive reappraisal, emotion regulation, and effective coping capacities. As a result, caregivers with higher resilience may rely less on external support, such as specialized care skill training and professional consultation, to enhance their caregiving competence, a pattern linked to a weaker incremental association between higher caregiving skills and lower depressive symptom scores. By contrast, caregivers with lower resilience typically possess fewer internal resources for emotional regulation and stress management, and may rely more heavily on external supports.

### 4.4. Limitations

This study has several limitations, which are mainly reflected in the following three aspects. Firstly, this study employed a cross-sectional design, which limits our ability to draw causal conclusions about the relationships between variables. Secondly, regarding the measurement of telehealth guidance service use, this study adopted a single measurement approach with a binary variable. Specifically, we only assessed whether respondents had ever used telehealth guidance services, and did not further explore the frequency-related characteristics of use (e.g., number of sessions, duration per use, regularity of use) and specific types of use. Thirdly, the limited sample size precluded a classified analysis of the utilization of diverse telehealth guidance services. As a result, it becomes challenging to precisely determine which specific form of telehealth guidance service significantly influenced the research outcomes. These shortcomings may further undermine the specificity and accuracy of the research conclusions.

## 5. Conclusions

This study provides empirical evidence for the associations between telehealth guidance service use and depressive symptoms, as well as the mediating role of caregiving competence and the moderating role of psychological resilience, among family caregivers of older adults with cognitive impairment.

The findings confirm that telehealth guidance service use was directly negatively associated with depressive symptoms, and was also indirectly negatively associated with depressive symptoms via caregiving competence. Meanwhile, psychological resilience significantly moderated the association between caregiving competence and depressive symptoms, with a stronger negative association observed among caregivers with lower psychological resilience. Collectively, these results highlight the complementary associations of external digital support and internal psychological resources with caregiver mental well-being.

Consistent with the low utilization rate of telehealth guidance services observed in this population, future service design may be tailored to the actual care needs of this group and adapted to real-world care settings to support greater uptake. Aligned with the study’s observed associations, telehealth programs may integrate both caregiving competency-building training and psychological resilience-focused components. These findings carry important implications for the design of evidence-based, person-centered digital health support, and offer practical reference for healthcare practitioners and policymakers to support the psychological well-being and long-term care quality for this vulnerable group of family caregivers.

## Figures and Tables

**Table 1:** Sociodemographic data, caregiving characteristics and depressive symptoms of family caregivers of older adults with cognitive impairment.

Variables	n	Percentage (%)	Depressive Symptoms, M (SD)	t/F	*p*
Gender				0.007	0.929
Male	190	38.70	9.68 (1.50)		
Female	301	61.30	9.69 (1.43)		
Ages				8.559	0.000
<45	102	20.77	9.20 (1.33)		
45–59	285	58.04	9.77 (1.45)		
≥60	104	21.18	9.98 (1.50)		
Educational level				1.139	0.321
Junior high school or below	185	37.68	9.82 (1.72)		
Senior high/technical secondary school	132	26.88	9.64 (1.46)		
Junior college or above	174	35.44	9.60 (1.32)		
Monthly family income (RMB)				6.123	0.000
≤5000	56	11.41	10.34 (1.66)		
5001–10,000	163	33.20	9.71 (1.57)		
10,001–20,000	165	33.60	9.70 (1.42)		
>20,000	107	21.79	9.33 (1.07)		
Daily caregiving duration				36.153	0.000
<1 h	119	24.24	8.70 (0.76)		
1–4 h	155	31.57	9.51 (1.07)		
5–8 h	136	27.70	10.16 (1.37)		
9–12 h	45	9.16	10.63 (1.56)		
>12 h	36	7.33	10.85 (2.36)		
Weekly caregiving days				15.413	0.000
1–2 days	272	55.39	9.32 (1.21)		
3–4 days	42	8.55	9.98 (1.46)		
5–6 days	164	33.40	10.16 (1.52)		
7 days	13	2.65	10.67 (2.67)		
Care recipient cognitive severity				110.468	0.000
Mild	319	64.97	9.14 (0.99)		
Moderate	145	29.53	10.47 (1.44)		
Severe	27	5.50	12.03 (1.92)		

Note: M = mean; SD = standard deviation. The *t*-test was used for gender, and one-way ANOVA was adopted for other variables.

**Table 2:** Descriptive statistics and bivariate correlations among study variables (N = 491).

Variables	M ± SD/n (%)	1	2	3	4
1. Telehealth guidance service use	85 (17.31)	1.000			
2. Caregiving competence	3.04 ± 0.48	0.218 **	1.000		
3. Psychological resilience	27.11 ± 7.54	0.154 **	0.188 **	1.000	
4. Depressive symptoms	9.69 ± 1.46	−0.337 **	−0.474 **	−0.317 **	1.000

Note: ** *p* < 0.01.

**Table 3:** Regression coefficients for the mediation model (N = 491).

Dependent Variable	Independent Variable	R	R^2^	F	β	t
Depressive symptoms	Telehealth guidance service use	0.669	0.448	56.003 **	−0.641	−4.669 **
Caregiving competence	Telehealth guidance service use	0.349	0.122	9.580 **	0.389	3.113 **
Depressive symptoms	Telehealth guidance service use	0.730	0.533	68.662 **	−0.474	−3.709 **
	Caregiving competence				−0.430	−9.341 **

Note: ** *p* < 0.01. Controls included age, monthly family income, daily caregiving duration, weekly caregiving days, and care recipient cognitive-impairment severity.

**Table 4:** Total, direct, and indirect effects in the mediation model.

Effects	β	SE	LLCI	ULCI	Percentage (%)
Total effect (X → Y)	−0.641	0.137	−0.911	−0.371	—
Direct effect (X → Y)	−0.474	0.128	−0.725	−0.223	73.95
Indirect effect (X → M → Y)	−0.167	0.055	−0.283	−0.064	26.05

Note: CI = confidence interval; LLCI = lower-limit confidence interval; ULCI = upper-limit confidence interval; X = telehealth guidance service use; M = caregiving competence; Y = depressive symptoms.

**Table 5:** Regression results for the moderated-mediation model (N = 491).

Variables	Regression 1 Outcome Variable: Caregiving Competence			Regression 2 Outcome Variable: Depressive Symptoms		
	β	SE	t	β	SE	t
Telehealth guidance service use	0.389	0.125	3.113 **	−0.417	0.127	−3.292 **
Caregiving competence				−0.366	0.051	−7.228 **
Psychological resilience				−1.193	0.375	−3.180 **
Caregiving competence × psychological resilience				−0.230	0.106	−2.172 *
R^2^	0.122			0.550		
F	9.580 **			58.633 **		

Note: * *p* < 0.05; ** *p* < 0.01. Controls included age, monthly family income, daily caregiving duration, weekly caregiving days, and care recipient cognitive-impairment severity.

**Table 6:** Conditional indirect effects at low and high levels of psychological resilience.

Psychological Resilience	β	SE	LLCI	ULCI
Low psychological resilience	−0.233	0.081	−0.406	−0.087
High psychological resilience	−0.143	0.049	−0.245	−0.053

## Data Availability

The datasets generated during the current study are available from the corresponding author on reasonable request.
